# Foix-Alajouanine Syndrome: A Case Report

**DOI:** 10.7759/cureus.36696

**Published:** 2023-03-26

**Authors:** Jorge del Pino-Camposeco, Eliezer Villanueva-Castro, Juan Antonio Ponce-Gómez, Sergio Ramírez-Aragón, Alan Hernández-Hernández, Juan Nicasio Arriada-Mendicoa

**Affiliations:** 1 Department of Neurosurgery, Instituto Nacional de Neurología y Neurocirugía Manuel Velasco Suárez, Mexico City, MEX

**Keywords:** spinal arteriovenous malformation, spinal dural arteriovenous fistula, foix-alajounaine syndrome, arteriovenous malformation, myelopathy

## Abstract

Foix-Alajouanine syndrome is a rare form of presentation of an arteriovenous malformation of the spinal cord that causes myelopathy in the thoracic and lumbar medullary segments. We present the case of a 46-year-old female who suffered from weakness in the lower limbs with sensation loss, low back pain, urinary incontinence, and constipation. The magnetic resonance image T2 sequence of the thoracic spine from T6 to T11 revealed abnormally hypointense signals in the posterior epidural region caused by larger arteries. A spinal digital subtraction angiography was useful to diagnose a right perimedullary fistula with venous drainage, which was satisfactorily embolized. The key to suspecting this diagnosis is the presence of dilated vessels in the posterior epidural space, which are evident in T2 and short tau inversion recovery (STIR)-weighted sequences. Physicians often misdiagnose Foix-Alajouanine syndrome, resulting in potential delays in care. Neurosurgeons can use surgery or endovascular embolization to treat this condition.

## Introduction

The Foix-Alajouanine syndrome was first identified in 1926 by two French neurologists, Charles Foix and Théophile Alajouanine. It is a myelopathy caused by a dural arteriovenous malformation (DAVM) of the spinal cord. It has a progressive evolution, and the spinal cord regions mainly affected are the lower thoracic and lumbar. Males are more commonly predisposed to this condition than females, with a ratio of 4:1. Most patients have a spinal dural arteriovenous fistula (SDAVF) in the lower thoracic region [1-3]. The lack of valves in the intraspinal venous system is the reason this region of the spinal cord is most susceptible to this DAVM. The physiopathology of this disease is not well understood, but an etiological hypothesis suggests that blood pressure elevations are transmitted into the dura mater by the arterial system through the dural venous system to the spinal venous plexus, which compromises the blood flow and leads to spinal cord ischemia. This may interfere with the spinal cord's typical drainage. Venous stasis, as opposed to thrombosis, is typically the primary cause of the infarction; this happens more in the late phase [1-3].

Foix and Alajouanine’s original description from autopsies showed spinal cord necrosis and multiple tortuous and thickened blood vessels on the surface of the spinal cord, later called necrotizing myelopathy [4]. Physicians often misdiagnose this condition, resulting in delayed offers of treatment and surgery [5,6].

A spinal arteriovenous malformation (SAVM) is an abnormal communication between a vein and an artery, also known as a shunt. They account for approximately 25% of all spinal vascular malformations and can manifest as both intramedullary and extramedullary [7]. These two types of SAVM are also classified into four categories: type I is an SDAVF with a single arterial feeder; type II is an arteriovenous malformation (AVM) of the intramedullary glomus type; type III refers to an extensive AVM with abnormal vessels, also known as a juvenile AVM; type IV is intradural direct arteriovenous fistula (AVF), consisting of a spinal and dilated perimedullary venous plexus direct fistula without a nidus in between. Type IV has three subdivisions: (i) just one nutrient artery with one small AVF with late ascending perimedullary venous drainage; (ii) a high-flow fistula with dilatation of the nutrient artery and draining vein; and (iii) multiple nutrient arteries connecting to a single giant AVF with profuse ectasic venous drainage [7-9].

The affected spinal segment has enlarged, tortuous, and thick-walled subarachnoid veins in the posterior region, as well as smaller, thicker-walled fibrotic blood vessels through the spinal cord [1]. These abnormal communications between arteries and veins are associated with venous reflux that produces venous congestion in the spinal cord, which results in venous hypertension and often leads to ischemic spinal cord injury [3]. Low diagnostic sensitivity in small AVMs results in a significant false-negative rate. Neurosurgeons cannot rule out the diagnosis of a spinal AVM even though the results of an initial angiography are negative or unclear. An early diagnosis of Foix-Alajouanine syndrome can facilitate the treatment of this condition and thereby improve the functional prognosis of the patients. Other authors recommend follow-up imaging with magnetic resonance imaging (MRI) and/or digital subtraction angiography (DSA) at appropriate intervals [9].

Here, we also describe a case of Foix-Alajouanine syndrome in a middle-aged female who presented for further evaluation. The aim of this report is to review the previously described characteristics of this disease as well as specific imaging findings.

## Case presentation

A 46-year-old female with no significant past medical and surgical history developed weakness in the lower limbs and moderate low back pain for four months, and a private office referred her to the National Institute of Neurology and Neurosurgery Manuel Velasco Suarez for further evaluation.

Physical examination showed diminished strength in lower limbs, with 2/5 in the right pelvic limb and 3/5 proximal and 2/5 distal in the left pelvic limb based on Daniel´s strength scale. She had a sensitive affection with hypoesthesia located in the T6 dermatome, patellar and Achilles stretch reflexes were poorly evoked; the rest was normal. She mentioned overflow incontinence and constipation with difficulty evacuating for one month of evolution.

**Figure 1:**
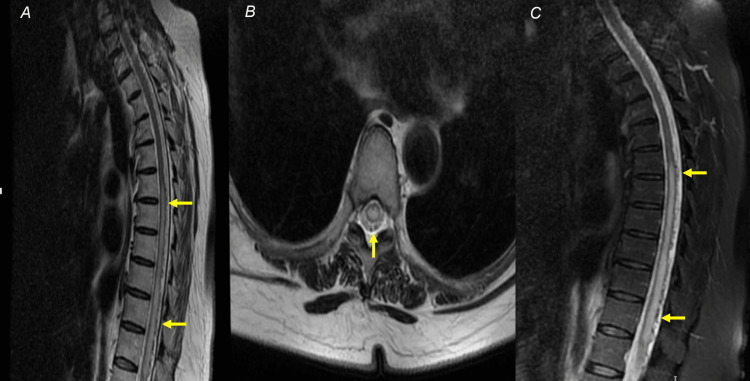
Spine magnetic resonance imaging. (A) Enlarged perimedullary vessels in the posterior epidural region in the T2 sequence (yellow arrows); (B) T2 sequences in the axial section occupy 80% of the cross-sectional area of the spinal cord with hypointense signal voids (yellow arrow; (C) STIR sequence with hyperintense signal image from T6 to T11 levels in the spinal cord caused by cytotoxic edema or myelomalacia (yellow arrows). STIR: short tau inversion recovery

**Figure 2:**
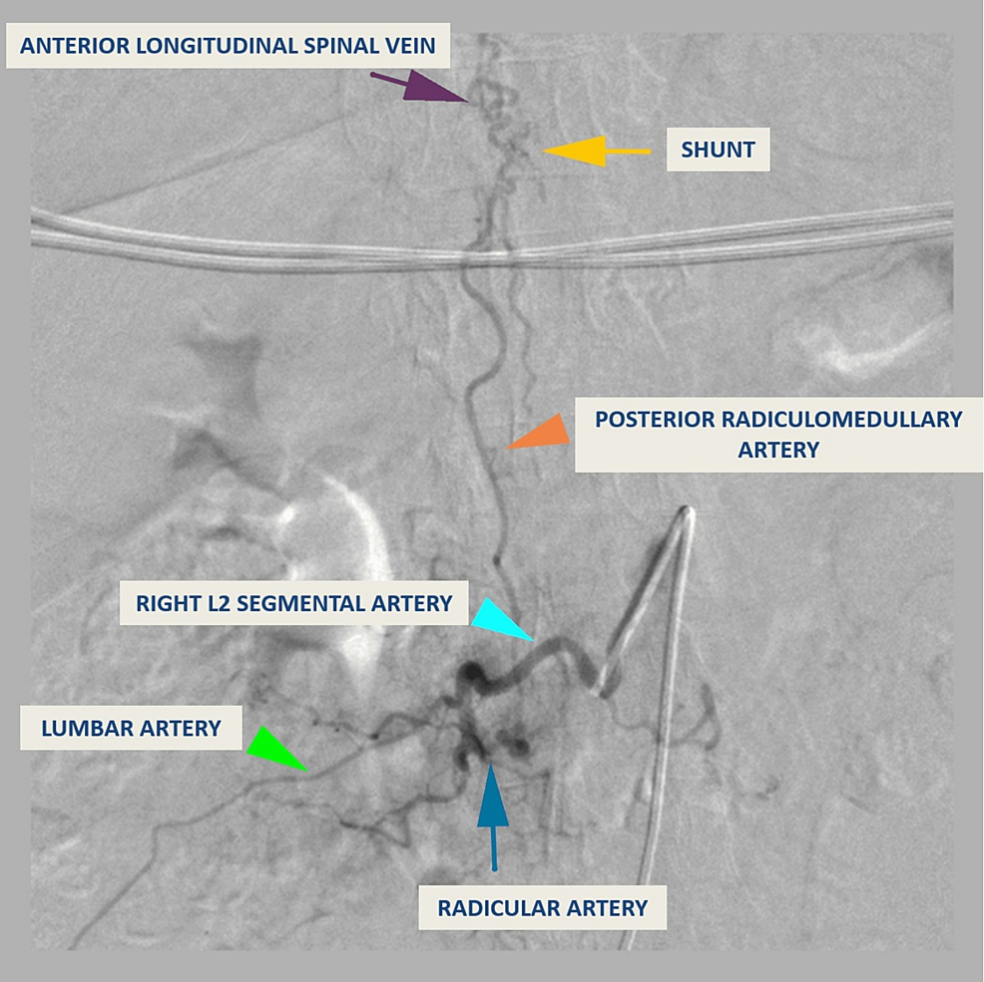
Spinal digital subtraction angiography. The spinal dural arteriovenous fistula (yellow arrow) is shown in conjunction with an afferent posterior branch in the posterior radiculomedullary artery (orange arrow) and a drainage spinal vein (purple arrow).

**Figure 3:**
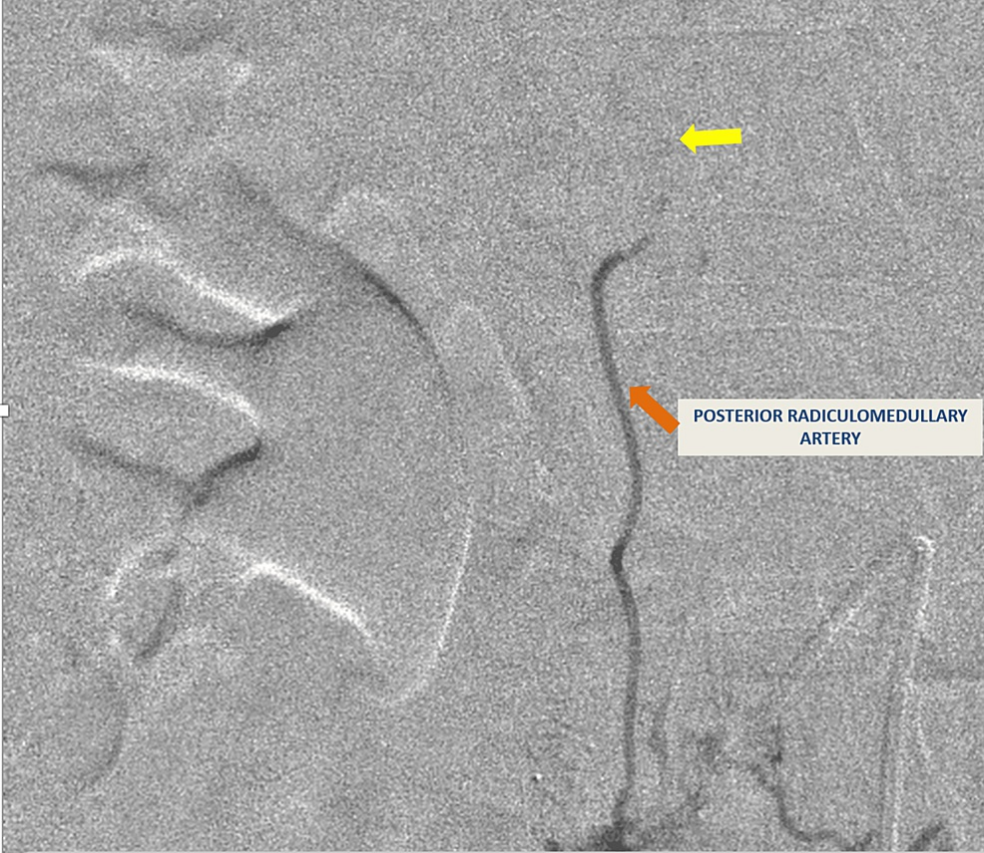
Spinal digital subtraction angiography after embolization. Shows the total closure of the perimedullary fistula (yellow arrow).

We ordered a spinal MRI, and the thoracic region showed a hyperintense signal image in the spinal cord from T6 to T11 levels in T2 and short tau inversion recovery (STIR) sequences, which occupied more than 80% of the circumference of the spinal cord in axial sections. This image was consistent with cytotoxic edema or myelomalacia. The image also showed enlarged perimedullary vessels in the posterior epidural region, with hyperintense signal voids in T1 and a hypointense signal in T2 sequences (Figure 1). Subsequently, we ordered a spinal DSA, and we found a right perimedullary fistula type I, with a feeding artery of the radiculopial branch and a perimedullary venous drainage of the segmental artery of the right L2 (Figure 2). It was satisfactorily embolized with a 20% histoacryl-lipiodol emulsion (Figure 3). Six months after the diagnosis and treatment of the present case, the patient was unable to walk due to a motor deficit similar to the previous one. 

## Discussion

Spinal vascular malformations are a diverse group of conditions that can present with acute, subacute, or chronic spinal cord dysfunction [10]. Vascular lesions in the spine represent 5-9% of central nervous system vascular malformations [11] and 3-4% of spinal intradural lesions [12].

The presentation of spinal AVMs can be acute or progressive; when it presents as an acute form, it is commonly associated with hematomyelia or subarachnoid hemorrhage, which is usually observed as intradural or intramedullary AVM and spinal cord aneurysms; the progressive presentation (secondary to venous hypertension, spinal cord ischemia, or mass effect) is more frequent in extradural or intradural AVF and AVM in the medullary cone and juvenile types. The majority of patients have some degree of motor or sensory deficit at the time of diagnosis [13]. In the presented case, the patient already had a motor and sensory deficit when she first came into contact with our service.

A venous stasis of the spinal cord caused by an arteriovenous malformation, leading to venous infarction and necrosis, is the cause of Foix-Alajouanine syndrome, a kind of acute or subacute myelopathy. The patients in the original study by Foix and Alajouanine had a type I AVF. These initial instances' pathological analyses revealed no signs of thrombosis, and the symptoms may have been caused by venous hypertension [4,14]. The patient in the described case had a type I dural fistula.

In spinal vascular lesions such as arteria-venous malformations, MRI findings can be normal in the early stages of Foix-Alajuoanine syndrome; however, when the disease progresses, the T1 sequence shows inflammation and hypointensity in peripheral regions of the affected spinal cord and, in the T2 sequence, central location hyperintensity in the spinal cord. It has been recommended that if spinal angiography shows no abnormality, an intracranial lesion must be looked for, as an intracranial AVF may cause myelopathy reflecting the transmission of venous pressure through the valveless Batson's plexus. In the phase-contrast MRI, areas of reinforcement and enlarged and tortuous vessels along the subarachnoid space could be observed, a phenomenon called "flow void" [1,2,5,7].

The treatment of choice for Foix-Alajouanine syndrome is either embolization by endovascular therapy, surgical ligation of the AVF, or both in some cases [15]. In our patient, endovascular therapy treatment was chosen following a meeting held in conjunction with the spine surgery service and neurological endovascular therapy. At first, these patients have spastic paraplegia, which eventually evolves into flaccid paralysis of the limbs with or without sphincter dysfunction. Later sequelae or terminal sepsis can lead to death. If patients don’t get treated before neurological symptoms occur, the prognosis is worse [6,15].

The progression of spinal vascular malformations, according to Aminoff and Logue, is progressive, with neurological deterioration and functional limitation. In their study, one-fifth of the 60 patients had to use crutches or were not able to walk after six months of the onset of symptoms other than pain. Fifty percent of the patients were in wheelchairs or bedbound after three years of the initial gait disturbance, and 91% had limited activity after three years of symptom onset [16].

It is common for some mild, transient deficits to occur after surgical or endovascular management, but this does not influence the short- or long-term outcome [17]. Authors may claim that the delay in diagnosis, rather than the severity of neurological damage, is the main cause of partial recovery because almost 48% of patients can experience illness progression before a diagnosis is made [10]. Patients who have decreased function as a result of an acute myelopathic episode will get some amount of symptom improvement post intervention if Foix-Alajouanine syndrome is correctly recognized and treated [17].

## Conclusions

Spinal vascular malformations are a rare entity, and they remain a diagnostic and therapeutic challenge. There are imaging findings in T1, T2, and STIR sequences that may suggest Foix-Alajouanine syndrome in MRI. Neurosurgeons should intentionally seek these to reach an early diagnosis and therapeutic approach. The duration between the development of the neurologic deficit and treatment can affect the patient's outcome.
